# Exposure to Biological Fluids in Dental Practice—Narrative Review on Appropriate Risk Assessment to Guide Post-Exposure Management

**DOI:** 10.3390/pathogens12070968

**Published:** 2023-07-24

**Authors:** Mihai Săndulescu, Mihnea Ioan Nicolescu, Cristian Funieru, Gülşen Özkaya Şahin, Oana Săndulescu

**Affiliations:** 1 Faculty of Dentistry, Carol Davila University of Medicine and Pharmacy, 020021 Bucharest, Romania; 2 Department of Translational Medicine, Faculty of Medicine, Lund University, 22362 Malmö, Sweden; 3 Laboratory Medicine, Department of Clinical Microbiology, Skåne University Hospital, 22242 Lund, Sweden; 4 Department of Infectious Diseases I, Faculty of Medicine, Carol Davila University of Medicine and Pharmacy, 021105 Bucharest, Romania; 5 National Institute for Infectious Diseases “Prof. Dr. Matei Balș”, 021105 Bucharest, Romania

**Keywords:** HBV, HCV, HIV, prophylaxis, post-exposure prophylaxis, dentists, healthcare workers

## Abstract

Accidental exposure to blood or other biological fluids is a common occurrence in dentistry, and its post-exposure management is a key component of infection prevention and control programs designed to prevent the transmission of blood-borne pathogens such as hepatitis B and C viruses (HBV, HCV) and human immunodeficiency virus (HIV). This narrative review aims to comprehensively review the risk assessment process for each of these pathogens at all steps of the epidemiological process, i.e., source–exposure route–receptive person, in order to provide a better understanding of the delicate differences that influence the transmission risk and that drive the individualized post-exposure management.

## 1. Introduction

Biological risk management is a key component of infection prevention and control in dental practice. When performing dental procedures, exposure to biological fluids is a frequent occurrence. For this reason, important measures are put into place worldwide to safeguard the health of dental practitioners, by minimizing the extent and the impact of this exposure in order to decrease the risk of acquiring blood-borne infections.

Pathogens such as hepatitis B and C viruses (HBV, HCV) and human immunodeficiency virus (HIV) are the most important blood-borne viruses responsible for chronic infections and are therefore the main focus of pre- and post-exposure prophylaxis for healthcare workers (HCWs). The risk of occupational acquisition of these blood-borne infections in dental practitioners requires careful consideration. A risk assessment should be performed both on a general level and on an individual level, by looking at two important indicators: the likelihood of the exposure event occurring and its consequences in terms of transmission odds, closely followed by an analysis of the likelihood of acquiring each specific infection and its consequences in terms of developing chronic infection.

## 2. Methods

For the purpose of this narrative review, we searched the medical and dental literature to identify the most recent information on the following four topics: (1) the rate of occurrence of accidental occupational exposure events in dentists and other dental healthcare personnel; (2) the dental care procedures most frequently associated with accidental exposure to blood or other bodily fluids; (3) the seroprevalence of blood-borne viruses such as HBV, HCV and HIV in different countries, in the general population and in particular risk groups; and (4) current recommendations for post-exposure prophylaxis interventions. The search was performed in April–May 2023 using PubMed to identify original articles to inform on items 1 through 3 in the list above and systematic reviews or national and international society guidelines to inform on item 4.

After analyzing the data from field literature as mentioned above and corroborating it with medical and virological information regarding the profile of the source patient, the route of exposure and the receptive person, the authors created an expert opinion section entitled “Factors influencing the rate of transmission of blood-borne viruses” and proposed an easy-to-use algorithm for individualized assessment of potential scenarios for risk transmission for each of the following blood-borne viruses: HBV, HCV and HIV.

## 3. Exposure to Biological Fluids among Dental Practitioners

To evaluate the likelihood of transmission of a blood-borne virus, a good understanding is needed of the rate of needlestick, sharp and splash injuries occurring in dental practice, as well as the seroprevalence of blood-borne viruses in the respective country.

The incidence of at least one event of accidental occupational exposure to blood has been reported to be at 26% among dentists and dental assistants in Lao within a 6-month time frame [1] and 29.2% among dentists in Saudi Arabia within a 12-month time frame [2]. When assessing the rate of occurrence of accidental professional exposure events at any time during the respondent’s career, comparable rates of 29.8% have been reported in dental assistants in Saudi Arabia [3], with higher rates of sharp injuries in Iran (54.5%) [4], and Croatia, where needlestick incidents were reported by 57.8% of dentists, cuts by 20.9% and eye conjunctival exposure by 13.4% [5]. In Poland, superficial injuries occurring during the delivery of dental care were reported by 60.4% of the dentists, while deep lacerations or needlestick injuries were reported by 16.7%; splashing of bodily fluids on the conjunctiva was reported by 54.7% of dentists, 28.1% reported mucous membrane exposure, while 27.6% reported exposure to bodily fluids through damaged skin during the past 12 months [6].

Percutaneous injuries reported by dental professionals in the USA from 1995 to 2001 occurred more frequently in dental assistants (75%), followed by dental hygienists (18%) and dentists (7%) [7].

Accidental exposure occurs as early as studenthood, with 60% of Egyptian dental school students reporting at least one needlestick injury during a surveyed interval of 6 months, and 38% reporting other sharp injuries [8]. Furthermore, historical data from the USA showed that from 1987 to 1997, dental students accounted for 82.1% of cases of accidental exposure in a dental teaching environment, while other dental staff accounted for 11.9% and teaching faculty for 6% [9].

The most important exposure-prone procedures in dentistry have been reported as follows: the disposal of needles or instruments (main cause for 37.2% of cases of exposure), performing or assisting sutures (22.3%) and the injection of anesthetic (17.9%) in a study performed in China [10]. Most of the percutaneous injuries reported among dental care professionals in the USA involved syringes (87%), suture needles (23%) and dental instruments (9%) [7]. A multicenter study performed in Sudan and Saudi Arabia showed that among dental school students, the three main procedures associated with percutaneous or conjunctival exposure included anesthesia (33.3–62.7% across different study sites), syringe use (29.3–55.6%) and the use of burs (37.4–77.8%) [11]. Among dental students in the USA, most instances of occupational exposure (54.5%) occurred post-operatively, predominantly during the process of instrument cleaning, while 41% occurred intra-operatively, during the use of the device [9].

## 4. Seroprevalence of Blood-Borne Viruses

The seroprevalence of HBV in the general population has been estimated by the European Centre for Disease Prevention and Control (ECDC) to range in Europe from as low as 0.1% for Ireland to as high as 3.3% for Greece and 4.4% for Romania, most European countries with available data reporting values of HBsAg positivity of around or below 1% [12].

For HCV, the historical (2016) antibody seroprevalence ranged from 0.1% in countries such as Belgium, Ireland and the Netherlands up to 3.2% in Romania and 5.9% in Italy [12]. These numbers most likely overestimate the current general burden of an HCV infection, for two reasons: (1) data regarding the confirmation of an active HCV infection by HCV-RNA was not available in the pooled ECDC estimates, and (2) these numbers predate to a large extent the widespread uptake of direct-acting antivirals (DAA) treatment for HCV infection in many of these countries. For instance, in a study that assessed outpatients presenting to a tertiary hospital with acute respiratory illness in Romania, the rates of HCV antibody (Ab) positivity have been reported at 2.9%, while the rates of positive HCV-RNA were much lower, reported at 0.2% of the total patient population [13].

For HIV, the seroprevalence in the European Union has been reported at 0.2% in the general adolescent and adult population aged 15–49 years, according to UNAIDS estimates provided by the World Bank [14]. The overall rate of new diagnoses among all persons tested for HIV in 2021 has been reported at 2.9% in recent years [15]. While the overall HIV seroprevalence is low, specific risk groups have higher rates; for example, recent European data showed a wide prevalence range in men who have sex with men (as low as 2.4% in Sweden up to as high as 29.0% in the Netherlands) and in people who inject drugs (ranging from 0% in Hungary to 59.5% in Estonia) [16]. Narrower ranges of HIV seroprevalence were reported among prisoners (from 0.04% in Hungary to 15.6% in Estonia), sex workers (1.1% in the United Kingdom up to 8.5% in Portugal) and transgender people (12.1% in Italy) [16].

## 5. Preventing Exposure to Bodily Fluids

The splash or splatter of saliva or blood are common occurrences during dental procedures. However, not each such episode poses a risk of transmission of blood-borne viruses. The correct wear of personal protective equipment (PPE) such as gloves, goggles and face mask provides a mechanical barrier to prevent transmission of infectious agents to dental practitioners during splash accidents. However, the wear of PPE is inconsistent, as seen from the field literature. For example, gloves were always used by 95% of the dentists included in a pre-COVID-19-pandemic study from Poland, but almost half of them also reported that sometimes they take off the gloves to facilitate certain dental procedures [6]. More worrisome, 6.5% of the dentists stated that they never use masks or goggles [6].

PPE use increased among dentists and other healthcare workers during the COVID-19 pandemic, due to a combination of contributing factors such as a heightened risk of transmission, a heightened perception of risk and PPE mandates [17]. As reported in Iran, the percentage of dentists who did not wear masks decreased from a pre-pandemic level of 4% to 0.8% during the beginning of the COVID-19 pandemic, the percentage of those who did not wear protective goggles decreased from 44.1% to 1.6%, and the percentage of those not wearing gloves decreased from 16.5% to 0% during the COVID-19 pandemic [18]. However, dental professionals also reported with a high frequency (84%) that COVID-19-mandated PPE was associated with decreased work efficiency and reduced visibility in the operating field (90%) [19]. For this reason, a decline in PPE uptake might have occurred after the compulsory measures during the pandemic were lifted, but hopefully, the use of masks, goggles and gloves will remain a part of standard dental practice going further. However, this remains a topic to be further explored, as no data is currently available regarding the post-pandemic PPE wear in the field of dentistry.

Even when used correctly, these protective barriers can break during the provision of direct dental care or during prosthetic or surgical maneuvers, particularly when needlestick or sharp injuries occur. The incidence of such occupational exposure is quite high, as described above, and is associated with very common dental procedures.

## 6. Factors Influencing the Rate of Transmission of Blood-Borne Viruses

**Figure 1:**
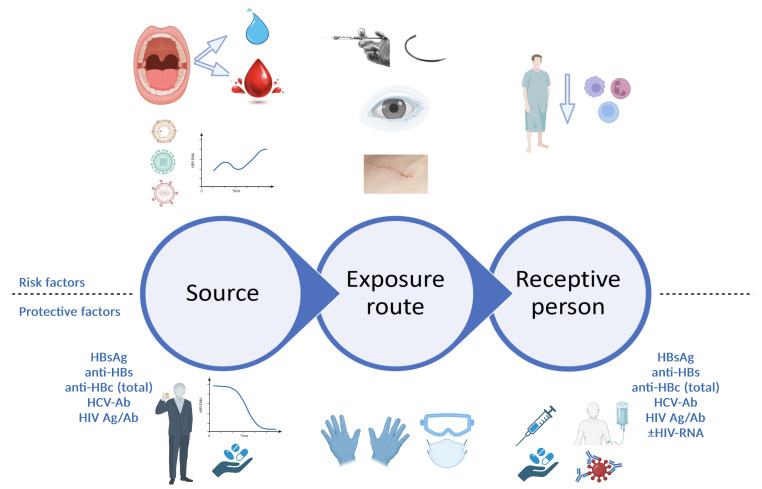
Risk assessment following professional exposure (figure created with BioRender.com). At each level of the epidemiological process, a series of risk factors and protective factors can be identified. When assessing the source patient, risk factors include undiagnosed or untreated active infection with viruses such as HBV, HCV or HIV, with high plasma viral loads, while protective factors include either absence of infection with blood-borne viruses altogether, or a prior diagnosis of viral infection with engagement in care and specific treatment, leading to suppressed viral loads for HBV and HIV and sustained virological response for HCV. For the exposure route, higher risks are associated with hollow needles, such as those used for anesthesia, and deep lacerations sustaining direct contact with large quantities of patient blood; correct use of appropriate personal protective equipment can reduce these risks. For the receptive person, risk factors include immunosuppression, lack of prior HBV vaccination or non-responsiveness to prior HBV vaccination, while protective factors include HBV vaccination with documented positive anti-HBs titer, as well as rapid initiation of PEP, with ART, HBV vaccination plus/minus HBV-specific immune globulins, as appropriate. HBsAg, hepatitis B virus surface antigen; anti-HBs, antibodies against hepatitis B virus surface antigen; anti-HBc, antibodies against hepatitis B virus core antigen; HIV Ag/Ab, human immunodeficiency virus antigen/antibody combined test; HBV, hepatitis B virus; PEP, post-exposure prophylaxis; ART, antiretroviral treatment.

The risk of transmission of a blood-borne virus following exposure is a direct product of each factor involved in the epidemiological process: source–transmission route (in this case, exposure route)–receptive population (in this case, receptive person) (Figure 1). A very important first step is to determine whether a significant exposure has indeed occurred. Therefore, the evaluation starts with an assessment of the exposure route, in order to decide whether further actions, such as source testing or post-exposure management, are warranted.

### 6.1. Assessment of the Exposure Route

The nature and the extent of exposure represent important drivers of the transmission risk. While certain exposures, i.e., the contact of saliva with intact skin, pose no transmission risk and do not warrant further action, exposure to biological fluids through broken skin, mucous membranes or, more importantly, percutaneous injuries carries a higher risk of transmission of blood-borne viruses and requires an in-depth assessment on all levels of the epidemiological process (source–exposure route–receptive person).

The highest risk is seen for percutaneous injury with direct contact with large quantities of patient blood (i.e., through deep lacerations induced by hollow needles) [20], followed by other types of exposure, such as the exposure of unprotected broken skin to patient blood or the mucosal/conjunctival splash of patient blood. Blood is by far the bodily fluid associated with the most important risk of transmission for HBV, HCV and HIV. However, other bodily fluids have also been studied, and this includes saliva.

The viral load for each different virus is much lower in saliva; for instance, only 68% of saliva specimens from patients with chronic HBV infection tested positive for HBV-DNA [21], and 7.2 log_10_ lower levels of HBV-DNA were reported in saliva compared to plasma in children with an HBeAg-positive chronic infection [22], while undetectable HBV-DNA was seen in saliva from children with an HBeAg-negative infection [22]. For HCV, 2.5 log_10_ lower HCV-RNA levels were reported in saliva compared to blood [23]. Among patients with HIV infection, only 29% of saliva samples were positive for HIV-RNA [24], and the levels of HIV-RNA in saliva were reported as 10% of plasma viral load levels [25], and most studies were performed in the pre-universal-ART era when a large proportion of the patients assessed were not on antiretroviral treatment. These lower viral load levels in saliva render the risk of transmission much lower; however, the risk is not null, as “zero risk” does not exist in medicine and biology, and it needs to be evaluated on a case-by-case basis and corroborated with information regarding the mechanism and the extent of exposure.

An additional important consideration is the fact that viral loads for HBV and HIV have been reported to be higher in the saliva of patients with periodontal disease [26,27], either through occult bleeding (reported to occur in 67% of patients with periodontal disease [27]) or through chronic inflammation and increased local endothelial permeability [28]. Furthermore, the gingival tissue has recently been added to the list of HIV reservoirs, at least in patients with marginal chronic periodontitis [26]. While generally interesting, this finding needs further studies with larger sample sizes to provide an understanding of whether or not this is also translated into a higher risk of transmission of HIV through saliva or if it is just an interesting pathology and histology finding.

### 6.2. Source Assessment

Whenever the source of the biological exposure is known, it is essential to obtain consent to test for HBsAg, anti-HBs, total anti-HBc, HCV-Ab (with reflex testing for HCV-RNA) and a combined HIV antigen/antibody test. A study from Iran showed that only 13.4% of dentists evaluated the source patient for hepatitis viruses and HIV [4], which is worrisome, since for each of these infections, different risks can be calculated based on different source patient profiles.

#### 6.2.1. HBV Infection

**Figure 2:**
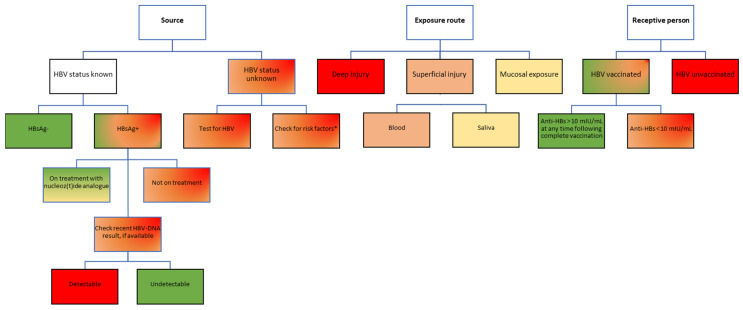
Risk assessment for HBV transmission following occupational exposure in healthcare workers. Color-coding legend: green boxes indicate the lowest risk; yellow to dark orange boxes indicate medium risk; red boxes indicate high risk. Gradient colors indicate varying degrees of risk, to be assessed based on individual factors. HBsAg, hepatitis B virus surface antigen; anti-HBs, antibodies against hepatitis B virus surface antigen; HBV, hepatitis B virus. * According to the USA Centers for Disease Control and Prevention recommendations, adults considered to be at high risk of HBV infection include people who inject drugs; people with multiple sexual partners, history of sexual contact with an HBV-infected person, men who have sex with men; unvaccinated household contacts of patients with chronic HBV infection; residents of long-term care facilities; prisoners; persons at risk for occupational exposure to HBV; patients on hemodialysis; persons with HCV infection; travelers to HBV-endemic countries; persons living with HIV; persons with diabetes [29]. Notably, persons from these categories only have a higher risk of testing positive for HBV; not all will be positive for HBV infection. These risk factors should only be used to temporarily guide the first PEP decision until an HBV result of the source patient becomes available, which will better inform decisions on further PEP steps.

A source with a chronic HBV infection can pose different levels of transmission risk, from low to high, based on whether or not the patient was aware of the diagnosis and engaged in specific care [29]. If a patient with an HBV infection has already been diagnosed and is undergoing treatment with a nucleot(z)ide analog, the HBV-DNA can be suppressed to undetectable levels, which lowers the risk of transmission to close to zero. If, however, the source patient has an undiagnosed HBV infection, not having been previously tested for HBsAg, there are two possible scenarios: (1) the patient has very high viral loads and poses a high transmission risk, or (2) the patient has relatively low levels of HBV-DNA and does not yet have an indication for treatment according to the local practice guidelines, which poses a moderate to low risk of transmission depending on the HBV-DNA level (Figure 2).

#### 6.2.2. HCV Infection

**Figure 3:**
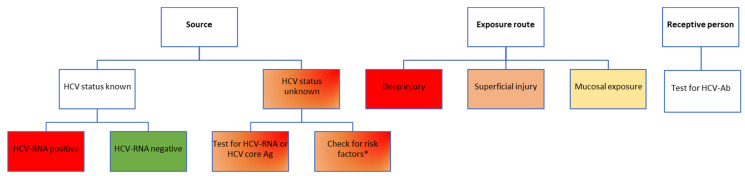
Risk assessment for HCV transmission following occupational exposure in healthcare workers. Color-coding legend: green boxes indicate the lowest risk; yellow to dark orange boxes indicate medium risk; red boxes indicate high risk. Gradient colors indicate varying degrees of risk, to be assessed based on individual factors. HCV-Ab, antibodies against hepatitis C virus; HCV-RNA, hepatitis C virus ribonucleic acid; HCV core Ag, hepatitis C virus core antigen. * According to the USA Centers for Disease Control and Prevention recommendations, adults considered to be at high risk of HCV infection include people who inject drugs [30]. This risk factor should only be used to guide the post-exposure testing recommendation for the healthcare worker until an HCV result of the source patient becomes available, which will better inform decisions on further testing.

A patient with positive HCV-Ab can either have an active infection (being positive for HCV-RNA and/or core antigen) or a prior history of a treated or spontaneously cleared HCV infection. The risks are also completely different in these different scenarios, as only an active infection can be transmitted (Figure 3). Unfortunately, most seroprevalence studies for HCV have only assessed HCV-Ab, and therefore, the real prevalence of active infection is not known in most settings at this point, several years following the introduction of universal DAA treatment for HCV infection, which has most likely led to an important decrease in the overall prevalence of HCV infection compared to pre-DAA estimates.

#### 6.2.3. HIV Infection

**Figure 4:**
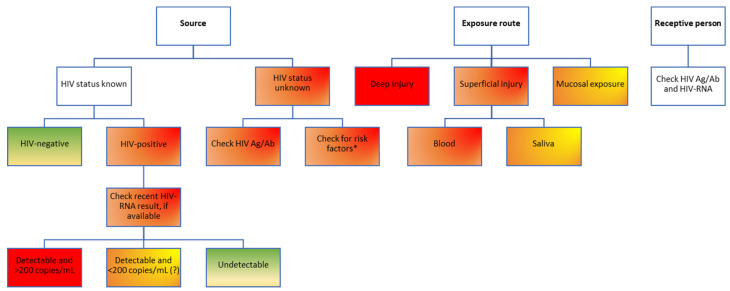
Risk assessment for HIV transmission following occupational exposure in healthcare workers. Color-coding legend: green boxes indicate the lowest risk; yellow to dark orange boxes indicate medium risk; red boxes indicate high risk. Gradient colors indicate varying degrees of risk, to be assessed based on individual factors. HIV Ag/Ab, human immunodeficiency virus antigen/antibody combined test; HIV-RNA, human immunodeficiency virus ribonucleic acid. * According to the USA Centers for Disease Control and Prevention experts, people considered to be at high risk of HIV infection include men who have sex with men; people who inject drugs; people with heterosexual contact with a person at risk for or infected with HIV; those with historical exposure to blood and blood products; persons at risk for occupational exposure to HIV [37]. Notably, persons from these categories only have a higher risk of testing positive for HIV; not all will be positive for HIV infection. These risk factors should only be used to temporarily guide the first PEP decision until a combined HIV antigen/antibody test result of the source patient becomes available, which will better inform decisions on further PEP steps.

Antiretroviral treatment is recommended for all patients with an HIV infection, regardless of the CD4 cell count, according to the European AIDS Clinical Society (EACS) guidelines updated in 2016 [31], and this is also true for other settings outside of the European Union, such as the USA. Theoretically, a person living with HIV who has been tested and is aware of the diagnosis is most likely already on ART and has, in most cases, a suppressed viral load. While strong data exist to prove that “undetectable equals untransmittable” (U=U) by sexual route for patients who are consistently virally suppressed, with HIV-RNA viral loads of <200 copies/mL [32], fewer data are available for the risk of transmission through blood and other fluids, with only sporadic case reports in the field literature [33]. From a theoretical standpoint, the lower the viral load, the lower the risk, according to the tried-and-true principle of “treatment as prevention (TasP)”, which clearly demonstrated more than 15 years ago that expanding antiretroviral treatment coverage has implications beyond the individual clinical benefit for each patient [34,35], being an important public health measure to decrease the number of new infections in the community [34,36]. Based on the profile of the source patient, three main risk categories can be described, listed in increasing order of the transmission risk: patients who have been diagnosed, are linked to care, receive antiretroviral treatment (ART) and are virologically suppressed; patients linked to care, on ART but with detectable viral loads (due to either recent initiation of ART or virological failure through low adherence or through resistance mutations); patients who have not yet been diagnosed, who have high viral loads and pose a high risk of transmission (Figure 4).

### 6.3. Assessment of the Receptive Person

Last but not least, the receptive person is another key factor to assess when considering the transmission risk. Safe and effective vaccines are available to prevent HBV infection [38,39], and most countries include newborn HBV vaccination in their national immunization plans (NIP). For example, Romania has included newborn HBV vaccination in its NIP since late 1995, and catch-up campaigns have subsequently expanded HBV vaccination to age cohorts dating back to the year of birth 1986. However, most of the adult working population in Romania has ages higher than 37 years old and has most likely not been vaccinated against HBV. It is therefore essential that after any kind of biological exposure, the status of the receptive person is also checked, and a triple panel is recommended, testing for HBsAg, anti-HBs, total anti-HBc, HCV-Ab (with reflex testing for HCV-RNA in case of a positive result) and a combined HIV antigen/antibody test, plus at least one HIV viral load measurement at baseline (Figure 1).

## 7. Tools for Post-Exposure Prophylaxis (PEP)

**Figure 5:**
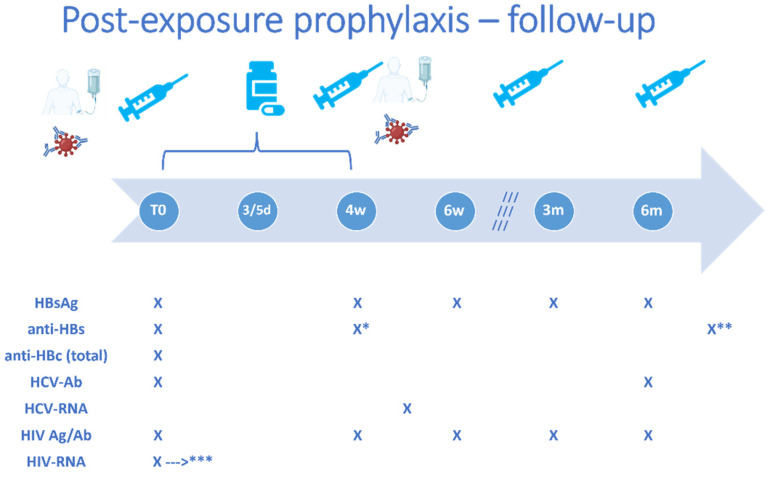
Important timepoints for post-exposure interventions and follow-up in healthcare workers. HBsAg, hepatitis B virus surface antigen; anti-HBs, antibodies against hepatitis B virus surface antigen; anti-HBc, antibodies against hepatitis B virus core antigen; HCV-AB, antibodies against hepatitis C virus; HCV-RNA, hepatitis C virus ribonucleic acid; HIV Ag/Ab, human immunodeficiency virus antigen/antibody combined test; HIV-RNA, human immunodeficiency virus ribonucleic acid; T0, baseline evaluation; d, days; w, weeks; m, months. The image depicts the timelines for administration of the following post-exposure interventions if indicated based on the individual risk assessment: HBV vaccine doses at 0–1–6 months or 0–1–2–6 months, as appropriate, specific anti-HBV immune globulins at 0 or 0–1 months, as indicated, and of antiretroviral post-exposure prophylaxis (for 28 days), as well as the timepoints when a laboratory follow-up workup should be performed: 4 weeks, 6 weeks, 3 months, 6 months. * anti-HBc will be repeated 4 weeks after an initial challenge/booster dose of HBV vaccine and ** 4 weeks after the complete vaccination regimen. *** HIV-RNA will be repeated in case any symptoms suggestive of acute infection occur during follow-up.

Based on the assessed risk level, different options for post-exposure prophylaxis will be recommended (Table 1, Figure 5). Following documented or suspected exposure to HBV or HIV infection, individualized PEP will be indicated for most cases considered to be at moderate to high risk. For HCV exposure, there currently is no recommendation for PEP, as pan-genotypic DAA treatment options are widely available and ensure high rates of sustained virologic response in case the transmission of HCV does occur [30], and in this case, the evaluation of the source and route of transmission will help tailor recommendations for the post-exposure testing of the healthcare practitioner. Specifically, for cases considered to be at medium to high risk of transmission, an HCV-RNA test should be performed 3–6 weeks after exposure, followed by HCV-Ab 6 months after exposure [30]. In case an active infection with HCV is documented, DAA treatment should be started promptly.

The decision for PEP following HBV exposure will be mainly based on the status of the receptive person. Specifically, if the HCW has been vaccinated with a complete three-dose series and has ever had an anti-HBs titer > 10 mIU/mL, no PEP is recommended, regardless of the status of the source and the magnitude of the risk calculated for the route of transmission [29]. However, even in this situation, in case the current anti-HBs titer is low (either < 10 mIU/mL or between 10–100 mIU/mL, depending on the clinical setting), certain practitioners would recommend a booster dose of the HBV vaccine. In HCWs with no/incomplete/unknown prior HBV vaccination, vaccination against HBV can be started immediately following exposure. This can either include a primary vaccination with a three-dose regimen in unvaccinated recipients (HCWs with undocumented vaccination status are considered unvaccinated for the purpose of deciding initial PEP management, according to the USA Centers for Disease Control and Prevention recommendations [29]) or as a single challenge/booster dose for those with a prior history of vaccination but with either unknown or negative (<10 mIU/mL) anti-HBs titers. In this latter case, a follow-up determination of anti-HBs should be performed approximately 4 weeks after the challenge/booster dose, to establish whether there is an indication to continue a complete three-dose regimen [29]. Furthermore, in cases considered to be at high risk, HBV-specific immune globulins can be co-administered with the first vaccine dose, ideally during the first 12 h following exposure, in order to confer protection until the person has time to mount an immune response following vaccination. A second HBV-specific immune globulin dose can be administered one month later for those at the highest risk of transmission, i.e., documented non-responders to the HBV vaccine after two complete three-dose series, having been exposed to an HBsAg-positive source patient with a detectable HBV viral load [29]. In recipients of HBV-specific immune globulins and the HBV vaccine, post-vaccination assessment of anti-HBs will be deferred for 6 months to avoid falsely elevated results [29].

For HIV, no prophylactic vaccine is available so far, but in the case of medium to high risk exposure, post-exposure prophylaxis with three antiretroviral agents should be considered, to be started as early as possible, ideally within the first 72 h following exposure, and continued for 28 days, with repeat testing for (at least) a combined HIV antibody/antigen test at 4 and 6 weeks and at 3 and 6 months post-exposure. Furthermore, in all cases where HIV PEP is indicated, plasma HIV-RNA should be assessed as well at baseline and, if negative, repeated if the HCW experiences any symptoms suggestive of acute infection during follow-up.

## 8. Discussion

Exposure to bodily fluids, particularly saliva, but also blood, is a common occurrence in dental practice, where the rate of percutaneous or mucocutaneous exposure has been reported to be very high. The frequency reported in the field literature varies between countries and settings, based on the timeframe set for data collection, i.e., certain studies have assessed 6-month incidence, others have assessed 12-month incidence, and others did not set a specific time frame. However, across all settings and timeframes, occupational exposure in dentistry is a frequent event, with at least one in four dentists experiencing an at-risk exposure to a biological fluid within 6 or 12 months of practice. This highlights the importance of understanding the complexity of the factors that go into the risk assessment process and that guide the decision of whether or what PEP to start.

Several protective measures have the potential to reduce the risk of acquiring infection following accidental occupational exposure. These include correct PPE wear, vaccination against HBV, immediate washing of the injured site with water and soap or irrigation with high volumes of normal saline of the conjunctiva/mucosa, within the first minutes after exposure to limit the contact time of patient bodily fluids with the broken skin/mucosa in order to reduce transmission risk.

An initial risk assessment should be performed by someone other than the exposed HCW, to ensure objectivity. Depending on each country’s regulations, infectious disease practitioners and/or occupational health practitioners are involved. This risk assessment should be undertaken for each individual episode of exposure to bodily fluids, because the driving factors (source, exposure route, receptive person) are slightly different in each case, requiring a tailored approach in terms of post-exposure management and follow-up.

To allow a rapid and accurate risk assessment, good communication between the dentist and the source patient is essential, in order to obtain consent to test for HBV, HCV and HIV or to acknowledge a prior diagnosis of infection with any of these viruses, and to share any available results of the most recent virological evaluation, particularly the viral load assessment, which can be a decisive factor for post-exposure prophylaxis recommendations.

If the source is unknown or unreachable, in case the overall risk of transmission is evaluated as high based on information regarding the exposure route and the receptive host, maximum coverage of post-exposure prophylaxis for HBV and HIV should be instituted to mitigate the risk as early as possible following exposure. Should further information about the source patients become available later, the initiated PEP can always be stopped or revised, as appropriate.

## 9. Conclusions

Dental procedures are associated with an important risk of exposure to bodily fluids. An individualized risk assessment should be performed for each case of exposure, in order to recommend the most appropriate approach to post-exposure prophylaxis. This includes an in-depth investigation of the sero-status of the source patient, an analysis of the risk posed by the exposure route and an evaluation of the receptive person, to check for susceptibility to HBV and to ascertain the baseline status for HBV, HCV and HIV, prior to initiating specific PEP procedures.

## Figures and Tables

**Table 1:** Options available for virus-specific pre- and post-occupational-exposure prophylaxis to prevent transmission of HBV, HCV and HIV. To be used in conjunction with standard precautions.

Virus	Pre-Exposure Prophylaxis Options	Post-Exposure Prophylaxis Options
**HBV**	Vaccination	PEP vaccination options: Primary vaccination * OR Challenge/booster vaccine dose ** OR N/A if evidence of response to prior vaccination exists HBV-specific immune globulins
**HCV**	N/A	Monitor and treat if infection occurs
**HIV**	N/A ***	ARV PEP

**HBV**, hepatitis B virus; **HCV**, hepatitis C virus; **HIV**, human immunodeficiency virus; **ARV**, antiretroviral; **PEP**, post-exposure prophylaxis; **PrEP**, pre-exposure prophylaxis. * In healthcare workers with negative history or unknown history of prior HBV vaccination, an accelerated primary vaccination regimen can be initiated post-exposure, with or without HBV-specific immune globulins, as based on the individualized risk assessment. In case of subsequent documentation of history of complete prior HBV vaccination with positive anti-HBs levels, the current vaccination regimen can be stopped and the first dose considered as a booster shot. ** In individuals previously vaccinated against HBV but without evidence of vaccine response, a challenge dose of HBV vaccine should be administered promptly. Anti-HBs levels should be checked at baseline and 4 weeks after this challenge dose—these results will inform whether or not subsequent vaccine doses are needed. *** ARV PrEP is available and efficient in decreasing sexual transmission of HIV in high-risk groups, but given the relatively low rate of exposure to HIV in clinical settings, healthcare workers are not considered to be a specific high-risk group, and PrEP has not been studied in people at risk of percutaneous exposure.

## Data Availability

The data supporting this article are publicly available in international databases. No new dataset was generated for the purpose of this article.
